# Unmet Needs of Artificial Intelligence in Small Bowel Capsule Endoscopy

**DOI:** 10.3390/diagnostics15091092

**Published:** 2025-04-25

**Authors:** Stefania Piccirelli, Daniele Salvi, Cecilia Lina Pugliano, Enrico Tettoni, Antonio Facciorusso, Emanuele Rondonotti, Alessandro Mussetto, Lorenzo Fuccio, Paola Cesaro, Cristiano Spada

**Affiliations:** 1 Department of Gastroenterology and Endoscopy, Fondazione Poliambulanza Istituto Ospedaliero, 25124 Brescia, Italy; stefania.piccirelli@poliambulanza.it (S.P.); cecilia.pugliano@poliambulanza.it (C.L.P.); enrico.tettoni@poliambulanza.it (E.T.); paola.cesaro@poliambulanza.it (P.C.); 2 Department of Experimental Medicine, Università del Salento, 73100 Lecce, Italy; 3 Gastroenterology Unit, Valduce Hospital, 22100 Como, Italy; ema.rondo@gmail.com; 4 Gastroenterology Unit, Santa Maria delle Croci Hospital, 48121 Ravenna, Italy; alessandro.mussetto@auslromagna.it; 5 Gastroenterology Unit, University of Bologna, 40136 Bologna, Italy; lorenzo.fuccio@unibo.it; 6 Digestive Endoscopy Unit, Fondazione Policlinico Universitario Agostino Gemelli IRCCS, 00168 Rome, Italy; cristiano.spada@policlinicogemelli.it; 7 Department of Translational Medicine and Surgery, Università Cattolica del Sacro Cuore, 00168 Rome, Italy

**Keywords:** bleeding, machine learning, diagnosis

## Abstract

Small bowel capsule endoscopy (SBCE) has emerged in the past two decades as the cornerstone for assessing small bowel disorders, and its use is supported by several guidelines. However, there are several limitations, such as the considerable time required for gastroenterologists to review these videos and reach a diagnosis. To address these limitations, researchers have explored the integration of artificial intelligence in the interpretation of these videos. In our review, we explore the evolving and emerging role of artificial intelligence in SBCE and examine the latest advancements and ongoing studies in these areas, aiming at overcoming current limitations.

## 1. Introduction

Small bowel capsule endoscopy (SBCE) has emerged in the past two decades as the cornerstone for assessing small bowel disorders. Recognized for its minimal invasiveness and high diagnostic accuracy, international societies recommend SBCE as the initial approach to investigate suspected small bowel bleeding (SSBB) and support its first-line use in case of suspected or established Crohn’s disease (CD) [1]. Despite its widespread adoption for several other indications and the enthusiasm surrounding recent technological advancements, SBCE continues to face several limitations. The most significant challenge remains the considerable time required for gastroenterologists to review SBCE videos and reach a diagnosis. A single examination generates tens of thousands of frames, requiring between 30 and 120 min to analyze [2,3]. This prolonged review time increases the risk of reader fatigue, which may lead to the oversight of critical findings. Moreover, detection, characterization, and recognition of lesions during CE reading are challenging, leading to suboptimal inter- and intra-observer agreements [4]. Additionally, precise lesion localization within the small bowel and accurate quantification of mucosal visualization during capsule transit remain unresolved challenges, making it difficult to reliably assess bowel cleanliness.

To address these limitations, researchers have explored the integration of artificial intelligence (AI) in the interpretation of SBCE videos. Early AI systems employed relied on conventional machine learning (ML) algorithms designed to streamline video analysis by identifying relevant frames and eliminating redundant or insignificant ones. While ML-based models have demonstrated acceptable diagnostic accuracy over the past decade, they have not yet proven sufficient to replace conventional reading [5]. However, the promising results paved the way for implementing AI models. Convolutional neural networks (CNNs) allowed revolutionary achievements of AI tools in dramatically reducing video review time and automating lesion detection [6]. If findings from the first prospective multicenter real-life study translate into clinical practice, AI-assisted reading for suspected bleeding lesions might achieve a diagnostic accuracy comparable to conventional methods while being nearly nine times faster [6]. In recent years, further advancements in lesion characterization have been reported [7,8,9]. However, significant challenges persist, particularly in lesion localization and the automated assessment and scoring of mucosal cleanliness.

In this narrative review, we explore the evolving and emerging role of AI in SBCE, examining the latest advancements and ongoing studies in these areas aiming at overcoming current limitations.

## 2. Materials and Methods

In our review, we conducted a comprehensive search across PubMed, Scopus, and Medline, including only English-language articles published up to the end of March 2025. Our search strategy utilized detailed search strings incorporating the following terms: “artificial intelligence”, “deep learning”, “machine learning”, “convolutional neural networks”, “small bowel capsule endoscopy”, “wireless capsule endoscopy”, “video capsule endoscopy”, “detection”, “characterization”, “localization”, and “cleansing”. Additionally, we manually screened the references of the included studies and relevant reviews to identify any further eligible publications.

## 3. The Role of Artificial Intelligence in Detecting and Characterizing Small Bowel Lesions

The characterization of small bowel lesions in terms of type, size, clinical significance, and location remains largely dependent on the subjective assessment of SBCE readers. Achieving consensus among gastroenterologists, regardless of their experience level, has proven to be a persistent challenge. A recent systematic review and meta-analysis conducted by Cortegoso Valdivia et al. [4] reported an overall pooled estimate of only 23% for “perfect” or “good” inter-observer agreement, with significant variability across studies depending on the statistical methods used.

This challenge extends beyond inter-observer variability, as intra-observer agreement is also suboptimal. In the same systematic review, only 37% of cases demonstrated “perfect” or “good” agreement when interpreted by the same observer. The heterogeneity of small bowel lesions further complicates interpretation, making accurate lesion characterization markedly more difficult than the detection of mucosal abnormalities. Since the introduction of SBCE in the early 2000s, efforts have been made to enhance the reproducibility of SBCE examinations. These efforts began with the Saurin classification for vascular lesions and their bleeding potential in 2003, followed by the Capsule Endoscopy Structured Terminology (CEST) classification in 2005, which aimed to standardize the SBCE report [10,11]. With the increase of SBCE application in the field of Crohn’s disease, scores such as the “Capsule Endoscopy Crohn’s Disease Activity Index” (CECDAI) and the Lewis score were developed to grade and quantify the severity of SB findings in patients with CD [12]. However, the interpretation of inflammatory lesions remained inconsistent, prompting a Delphi consensus published in 2020 to standardize nomenclature and descriptions for ulcerative and inflammatory lesions [13].

Among the most challenging aspects of SBCE interpretation is distinguishing submucosal masses from benign bulges. A retrospective multicenter study assessed the accuracy of various scoring (SPICE, Mucosal Protrusion Angle -MPA-, SHYUNG) in differentiating subepithelial masses from bulges. While these scores are relatively simple to apply during SBCE reading, they demonstrated poor inter- and intra-observer agreement, with kappa values ranging from 0.14 to 0.44. Sensitivity was limited, ranging from 18.7% to 58.7%, despite a specificity of 76% to 92% [14].

In general, tasks that are difficult for human readers tend to pose even greater challenges for AI systems, necessitating extensive training. Over the past seven years, AI systems have demonstrated remarkable accuracy in the automated detection of vascular and inflammatory lesions, achieving sensitivity between 89.5% and 100% and specificity between 96% and 98.4% [15,16,17,18,19,20,21,22]. However, much of the reported performance is based on retrospective studies or assessments using carefully selected datasets, rather than evaluations conducted in routine clinical full-length videos. Consequently, although AI systems have shown accuracy comparable to, or even exceeding, that of human readers in experimental conditions, they have yet to demonstrate the consistent reliability required to substitute conventional interpretation in everyday medical practice. Automated lesion characterization, in particular, faces challenges in achieving optimal performance.

Since 2021, several CNN systems have been tested for lesion characterization, for potentially bleeding and ulcerative lesions. In a proof-of-concept study, Hwang et al. [23] proposed a combined CNN-based model for classifying hemorrhagic and ulcerative lesions without manual annotations achieving a sensitivity of 97.6%. Similarly, the SUM-UP study evaluated the performance of a deep learning algorithm combined with a random forest algorithm to detect and define specifically vascular lesions with high to moderate bleeding potential (P2 and P1 according to the Saurin classification, respectively). The combined system achieved a 98% agreement with conventional (human) reporting in terms of selection of the most relevant frame within sequences with vascular lesions, within a mean time of 20 s per full-length small bowel video [9].

The multicenter retrospective study of Mascarenhas Saraiva et al. [24] validated a CNN system for detecting and classifying vascular lesions based on the Saurin classification. The system achieved high sensitivity (88%, 87%, and 94%) and specificity (100%, 97%, and 99%) for P0, P1, and P2 lesions, respectively. In another multicenter retrospective study, Aoki et al. [25] compared a new CNN system with the QuickView mode of the RAPID CE reader software (Medtronic, Minneapolis, MN, USA). When assessing the CNN system’s ability to diagnose small bowel lesions—including mucosal breaks, angioectasias, protruding lesions, and blood content—compared to expert SBCE readers, the AI model outperformed QuickView (99% vs. 89% per-patient detection rate). Concordance rates between the CNN system and expert readers were 95.7% for mucosal breaks, 75.9% for angioectasias, 98.8% for protruding lesions, and 100% for blood content.

**Figure 1:**
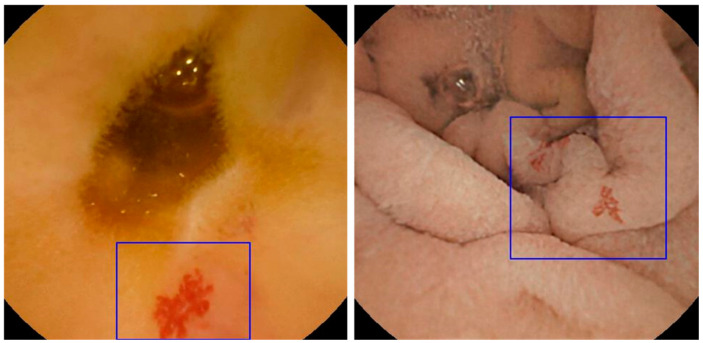
ANKON system—ProScan function: Automated detection of jejunal angiectasia, with a blue box highlighting the lesion(s).

**Figure 2:**
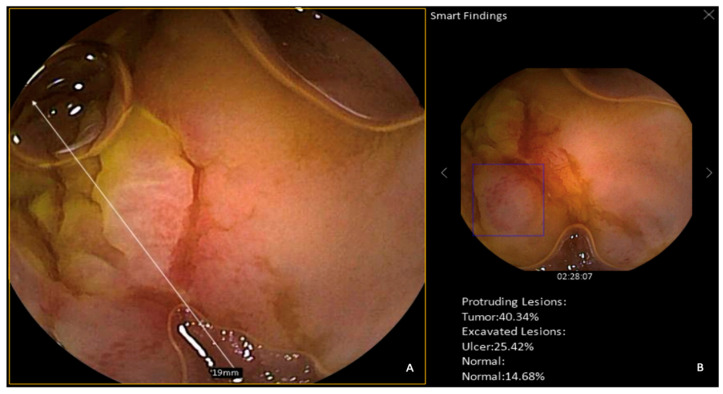
OMOM system—(**A**) Ileal erosion. (**B**) SmartScan function: automated detection of the lesion, with a blue box highlighting the affected area. Smart Finding function: lesion characterization (prediction).

At the current state of the art, only two SBCE systems embedded with a CNN tool capable of automatically detecting small bowel abnormalities are available in Europe: the Ankon technology, integrated with ProScan (AnX Robotica, Plano, TX, USA), and the OMOM Capsule Endoscopy System, equipped with SmartScan (Chongqing Jinshan Science & Technology Co., Ltd., Chongqing, China) (Figure 1 and Figure 2). However, no prospective studies using full-length videos have yet been conducted to evaluate their performance in lesion characterization. This highlights the need for further research focused on this area, particularly to support the integration of these tools into routine clinical practice and to provide robust evidence for regulatory authorities.

Ding et al. validated an advanced version of ProScan (Ankon Technology, Macedon, NY, USA) capable of recognizing eight distinct small bowel lesion categories—red spots, inflammation, blood content, vascular lesions, protruding lesions, parasites, diverticula, and normal variants—using 240 retrospectively collected videos [7]. In the per-lesion analysis, the model achieved an overall accuracy of 89.4%, with performance ranging from 79.2% for vascular lesions to 97.5% for parasites. Additionally, the AI model significantly enhanced the diagnostic performance of junior readers. Compared to conventional reading, AI-assisted reading improved diagnostic accuracy from 85.5% to 97.9% and sensitivity from 65.9% to 99.2%, even surpassing the accuracy of conventional reading by experienced gastroenterologists (overall accuracy 96.6%) (Figure 1).

Xie et al. evaluated the performance of an updated version of SmartScan, SmartScan Plus (SSP, Chongqing Jinshan Science & Technology Co., Ltd., Chongqing, China), in recognizing five types of gastric lesions (erosion, ulcer, redness, polyp, blood) and 17 types of small bowel lesions (aphtha, erosion, ulcer, nodule, lymphangiectasia, venous structure, polyp, parasite, blood, erythematous, edematous, abnormal villi, white plaque, red plaque, red spot, angiectasia, mass/tumor) using 342 videos retrospectively collected [26]. The study compared the performance of junior and expert endoscopists in lesion detection with and without SSP assistance, confirming previous findings that AI-assisted reading is nearly ten times faster than conventional reading and significantly enhances diagnostic yield, particularly for junior readers (diagnostic yield for small bowel lesions increased from 89.47% to 97.66%), effectively bridging the gap between trainees and expert readers. However, Xie et al. did not measure SSP reliability in automated characterization (“Smart findings” function) of any small bowel type of lesion. (Figure 2).

As a result, even if the SSP function that characterizes lesions with a certain grade of prediction is currently included in the OMOM system, it cannot be confidently used in clinical practice. Similarly, automated lesion characterization by ProScan (Ankon technology) still needs prospective clinical validation to be embedded in the current available Navicam system.

## 4. The Role of Artificial Intelligence in Assessing Small Bowel Cleanliness

To attain a high diagnostic yield during SBCE, it is crucial to ensure adequate small bowel preparation in a minimum of 80% of procedures [27]. However, the best preparation before SBCE remains uncertain. In a recent meta-analysis, it was demonstrated that the administration of a purgative solution was superior to only fasting, and better cleansing was obtained when bowel preparation was given closer to the ingestion of the capsule [28]. Following the European Society of Gastrointestinal Endoscopy, every report should include a detailed assessment of bowel cleansing quality using a validated scale to enhance the reliability of findings [29]. While there are numerous operator-dependent scales evaluating overall adequacy assessment and/or quantitative and/or qualitative parameters, the most widely adopted in clinical practice are the Brotz and Park scales [30,31,32]. To reduce the risk of subjectivity and lengthiness, several computer-dependent scales have been recently proposed.

In 2011, Van Weyenmberg et al. proposed the first computed assessment of cleansing (CAC) based on the red over the green (R/G) pixel index on the PillCam tissue color bar encompassing 10 cases [33]. The concept was grounded in the hypothesis that alterations in the tissue color bar could be indicative of enteric cleanliness. Subsequently, the CAC score was prospectively validated in 85 new cases and adapted for use with the MiroCam map view [34,35]. In both systems, there was a good correlation with classical scores but there was no clear definition of adequate mucosal visualization. To address this concern, a new CAC was developed and validated on PillCam SB2 in a carefully selected group of patients undergoing SBCE for obscure gastrointestinal bleeding (OGIB) [36]. As a result, a sensitivity of 91.3% and a specificity of 94.7 were achieved.

Notably, all these studies did not consider the presence of bubbles, and there was a lack of validated scales for assessing bubble numbers. Pietri et al. introduced an innovative computerized method to address this gap, relying on a grey-level co-occurrence matrix (GLCM) detector strategy. The algorithm achieved a sensitivity of 95.79% and a specificity of 95.19% [37].

The primary limitation of the previously discussed systems lies in their singular focus on evaluating a single aspect of cleanliness. To address this limitation, an innovative approach was adopted, combining a GLCM algorithm for bubble detection with an R/G ratio for cleanliness assessment and a brightness index. This multifaceted approach has enabled the development of a comprehensive and automated assessment method for evaluating mucosal visualization quality. The system demonstrated a sensitivity of 90.0% and a specificity of 87.7%, along with excellent reproducibility [38].

With the advent of CNNs, numerous novel algorithms have emerged. These systems have undergone training on extensive image datasets, encompassing various capsule types and diverse clinical indications. They have achieved remarkable levels of accuracy, spanning from 69.4% to 95.23% [39,40,41,42,43]. A concise summary of the CNNs currently available is provided in Table 1.

The adoption of automated small bowel evaluation scales represents a significant step forward in research efforts. These scales have the potential to facilitate the achievement of consensus on the optimal approach to assess bowel cleanliness. Such consensus has been elusive in the past due to conflicting results in studies, largely stemming from the absence of standardized assessment methods, especially regarding the timing between bowel preparation and the SBCE procedure. To this regard, Oh et al. conducted a study utilizing a validated CNN algorithm on SB3 to investigate whether there was a variance in small bowel cleanliness when SBCE was performed immediately after a colonoscopy compared to when it was conducted separately. Their findings demonstrated that the bowel preparation did not differ significantly whether SBCE was performed immediately after colonoscopy or as a standalone procedure [44].

Despite the significant advancements in SBCE technology, the lack of an AI-integrated clinically viable cleansing assessment remains a major barrier to its widespread integration into routine medical practice. The absence of such standardized tools limits the ability to consistently and objectively evaluate bowel cleansing quality, which is essential for accurate diagnostic interpretation. An automated cleansing assessment would also help define which is the best bowel preparation regimen for CE. Evidence on this topic is controversial, mainly because the comparison between different regimens has always been subjective. Moving forward, the development and implementation of robust, automated cleansing assessment solutions are imperative to enhance diagnostic precision, improve workflow efficiency, and ultimately optimize patient outcomes in SBCE.

## 5. Capsule Localization: A Critical Component in Small Bowel Capsule Endoscopy

Accurate localization of SBCE and its pathological findings within the gastrointestinal tract is critical for optimizing patient management. Determining the exact location of significant small bowel lesions is essential to guide subsequent diagnostic and therapeutic interventions. However, unlike conventional upper and lower endoscopy, SBCE lacks anatomical landmarks within the small intestine. The small bowel’s featureless, tubular structure allows the capsule to move freely in all directions, making lesion localization highly subjective and reader-dependent [45].

Furthermore, the inability to precisely determine the capsule’s location within the small intestine imposes significant limitations on future advancements, such as targeted drug delivery or tissue sampling. The next generation of SBCE systems may incorporate navigation assistance via external devices, such as magnetic or electromagnetic wave-based systems, or wireless power transmission to extend capsule battery life. These innovations could enable targeted small bowel exploration and allow complete enteroscopy in cases of slow transit time [46].

The current SBCE localization strategy is transit-time based, which approximates capsule position by measuring the time elapsed between passage through anatomical landmarks (e.g., stomach entrance, duodenum entrance, and cecum entrance). However, this approach has well-documented limitations in accuracy and remains an imprecise, reader-dependent technique [46]. To the best of our knowledge, an AI system called Smart Data Service System (SDSS-AI) studied by Jun Pan et al. is the only CNN model applied to automatically locate a capsule in the GI tract [47]. SDSS-AI was trained on 34,062 still images from 856 patients and later validated on an additional 50 patients, using expert reading as a gold standard. The system achieved a 94.2% accuracy in identifying various gastric anatomical landmarks, and a 98.9% sensitivity for detecting gastric lesions (98.7% sensitivity for gastric erosion, bleeding, and ulcers; 100% sensitivity for polyps and submucosal tumors). The landmark recognition accuracy ranges from 73.8% for the gastric body and 98.8% for the pylorus. Overall, SDSS-AI appears to be a promising tool for real-time diagnosis and localization of gastric lesions. Although these findings are encouraging, they were derived from a study conducted in the stomach, a relatively fixed anatomical environment. Consequently, further investigation is needed to evaluate the applicability and accuracy of a similar localization method within the more mobile and anatomically complex small bowel.

Several alternative localization methodologies have also been explored, including magnetic field-based, electromagnetic wave-based, and computer vision-based approaches.

Magnetic tracking systems offer several advantages in SBCE, including their ability to penetrate tissue, operate without a direct line of sight, and potentially enable capsule steering and controlled locomotion [45,46]. However, a key challenge of these systems is their susceptibility to external magnetic interference, which remains a major limitation requiring further refinement [46].

Radio wave tracking systems are integrated into many SBCE setups, where capsules are equipped with radio frequency (RF) transmitters for data transmission [48]. For instance, Nafchi et al. explored the use of circular antennas placed around the body to receive RF signals and calculate capsule position using conventional RF-based methods. However, their system exhibited localization errors of up to 10 mm [48]. Factors such as tissue density and organ arrangement significantly impact signal propagation, making RF-based localization less reliable compared to alternative methodologies [49]. Traditional imaging modalities, including X-ray, gamma-ray, magnetic resonance imaging (MRI), computed tomography (CT), and ultrasound, have not yet been successfully integrated with SBCE. Continuous imaging during capsule transit would expose patients to radiation risks, limiting their feasibility in routine clinical practice [48,50]. 

While SBCE localization techniques have evolved significantly in recent years, there remains a critical gap in the development of AI-integrated, commercially available tools for precise lesion localization in the small bowel. Further research in this area is essential, as AI-driven localization could transform patient management and significantly improve therapeutic outcomes.

## 6. Barriers to Clinical Integration of AI in SBCE

While AI has demonstrated considerable potential in SBCE, several limitations remain that preclude immediate routine clinical implementation. A primary concern is the generalizability of current AI models, many of which are trained on restricted datasets that may not encompass the full spectrum of variability or image quality encountered in daily practice. Suboptimal bowel preparation, luminal debris, and motion artifacts continue to present significant challenges, often leading to reduced accuracy in lesion detection.

Moreover, the majority of available performance data stems from retrospective analyses or short video segments. Validation through robust, prospective, and multicenter studies remains essential to assess real-world effectiveness. Importantly, regulatory authorities will require such evidence before approving tools for widespread use, particularly in settings where AI may assume a leading role.

In addition, unresolved issues regarding legal accountability and ethical responsibility persist, especially in the context of fully automated reading and diagnosis. These shared limitations across AI applications underscore that current technologies are not yet ready for fully autonomous deployment and highlight the need for structured prospective trials.

## 7. Conclusions

An AI system that truly enhances SBCE must extend beyond only lesion detection. An ideal system should not only identify relevant findings but also characterize them, localize their anatomical position, and determine their extent. Simultaneously, it must provide a clear and reliable assessment of bowel cleanliness, to ensure examination quality and diagnostic accuracy. Without the integration of these capabilities, AI cannot independently provide a comprehensive and clinically effective SBCE assessment in terms of accuracy, cleanliness, and overall diagnostic value.

Currently, no AI tool in clinical practice brings all these elements together. However, if we envision a future in which AI takes the lead in SBCE reading—under clinician supervision rather than through frame-by-frame manual review—then the development of a fully integrated system becomes essential. Future research should focus on the development and validation of unified models that incorporate the capabilities of currently separate AI tools within a single framework. Such an approach would promote consistency in SBCE interpretation, enhance inter-observer agreement, and support standardized clinical decision-making. This includes optimizing strategies for device-assisted enteroscopy and assessing disease extent and severity in Crohn’s disease, thereby guiding personalized therapeutic interventions.

Research into AI-driven lesion detection and bowel cleanliness assessment is progressing rapidly, with promising preliminary results. If future studies using full-length SBCE videos demonstrate that AI-assisted reading achieves diagnostic performance comparable to or exceeding that of conventional methods, these technologies are likely to become an integral part of routine clinical practice. Moreover, the incorporation of accurate capsule localization and navigational support could significantly enhance diagnostic efficiency and improve overall patient care.

## Figures and Tables

**Table 1:** Convolutional neural network-based algorithms currently available for the assessment of SBCE bowel preparation.

Author	Year	Capsule Type	Training Set	Indication	Scale Type	Accuracy
Noorda R. [39]	2020	Pillcam SB3	55,293 images	All indications	Qualitative	95.23
Leenhardt R. [40]	2021	Pillcam SB3	600 images	OGIB	OAA	89.7
Nam J.H. [42]	2021	Pillcam SB3	71,191 images	All indications	Quantitative	69.4
Nam J.H. [41]	2021	MC-1000 MC-1200 MC-4000	280,000 images	All indications	Quantitative	93
Riberio T. [43]	2023	Pillcam SB3 OMOM HD	12,159 images	All indications	Quantitative	92.1

MC: MiroCam; OGIB: Obscure gastrointestinal bleeding; OAA: Overall Adequacy Assessment.

## Data Availability

The review is based on the currently available literature.

## Abbreviations

The following abbreviations are used in this manuscript:

SBCE	Small bowel capsule endoscopy
SSBB	Suspected small bowel bleeding
CD	Crohn’s disease
AI	Artificial intelligence
